# Social behaviors and HIV risk factors among men in Chad and Cameroon

**DOI:** 10.11604/pamj.2021.38.331.27237

**Published:** 2021-04-06

**Authors:** Bonheur Dounebaine, Kate Winskell

**Affiliations:** 1 Rollins School of Public Health, Emory University, Atlanta, Georgia, United States

**Keywords:** Chad, Cameroon, Africa, HIV testing, sexual behavior, condom use, HIV knowledge

## Abstract

**Introduction:**

the neighboring Republics of Chad and Cameroon have respectively one of the lowest and highest HIV prevalence rates in central Africa at 1.3% and 4.5%, respectively. We conducted a comparative description of social behaviors and HIV risk factors among heterosexual men and explored the baseline of the first UNAIDS target (by 2020, 90% of people living with HIV will know their status) in the two countries.

**Methods:**

this was a retrospective cross-sectional study using Demographic and Health Survey data. We fitted a separate multilevel logistic model for each country. In total, 5248 men were interviewed in Chad and 7191 men in Cameroon.

**Results:**

Cameroonian men have a higher level of education, higher HIV testing rate, and are more knowledgeable about HIV than Chadian men. However, Chadian men have a lower number of lifetime sexual partners (2 interquartile range (IQR), 1-4) compared to Cameroonian men (6 IQR 3-15) and 86.96% of Chadian versus 57.30% of Cameroonian men reported fidelity to their domestic partners in the last twelve months.

**Conclusion:**

there is a crucial need to encourage HIV screening and testing among Chadian men, especially in rural areas. Testing also needs to be increased in Cameroon to meet the first UNAIDS target. Government and partners in Cameroon could support more research and campaigns that aim at reducing multiple sexual partnerships among the communities in Cameroon.

## Introduction

In 2014, the Joint United Nations Programme on HIV/AIDS (UNAIDS) set an ambitious goal of ending the HIV epidemic by 2030. To monitor progress, UNAIDS established the 90-90-90 targets to be achieved by all stakeholders and partners, whereby 90% of all HIV-positive persons will be diagnosed, 90% of those diagnosed will be on antiretroviral therapy (ART), and 90% of people receiving ART will have achieved viral suppression by 2020 [1]. The adjacent Republics of Chad and Cameroon, have respectively one of the lowest and highest HIV prevalence rates in the central African region. Chad and Cameroon have respectively a population of 15.4 million and 25.2 million people [2]. The most recent UNAIDS estimates of HIV prevalence rates were 3.6% and 1.3% respectively in Cameroon and Chad [3]. As in Chad, HIV transmission in Cameroon is mainly heterosexual [4]. Despite a higher HIV prevalence than Chad, the Republic of Cameroon has a stronger health care system and is better equipped regarding health care facilities and qualified human resources. There are approximately 1.1 physicians and 7.8 nurses and midwives per 10,000 population in Cameroon [5] (0.44 physicians and 3.09 nurses and midwives per 10,000 population in Chad [6]). A 1989 study that first reported the presence of HIV in Chad found a prevalence rate varying from 0 to 1.1% among regions [7] and the epidemic peaked in 2002 with a national prevalence rate of 3.4% [8]. However, according to the UNAIDS 2016 estimates, Chad is among the few countries that have reduced HIV incidence by more than 50%, from 2005 to 2015 [9]. The Chadian government has made significant efforts to strengthen HIV control and prevention: the Ministry of Health (MoH) implemented free access to antiretroviral therapy (ART) and care since 2007; created regional committees on HIV in 17 or 23 regions; created a budget line for the national committee on HIV (Conseil National de Lutte Contre le SIDA, CNLS); and since 2012 there was an expansion of elimination of mother to child transmission (e-MCT) services [10]. Additionally, international partners including UNICEF, WHO, and UNFPA have played a major role in supporting the MoH. However, the Chadian health system suffers from multiple deficits, including insufficient medical staff and inequitable distribution of health workers across provinces. The epidemic is disproportionately distributed among regions; the southern regions bore the highest burden with 5.55% prevalence in 2011 [10], which is in part due to the proximity to Cameroon and the Central African Republic (CAR) whose national HIV prevalence rates are higher (4.5% and 3.7% respectively) [11].

In Cameroon, the first case of HIV was reported in 1986 [12], and the prevalence peaked at 5.4% in 2000 [13]. According to UNAIDS 2016 estimates, there has been a 25-49% increase in HIV incidence among adults (aged 15 years and older) in Cameroon from 2005 to 2015 [9]. Cameroon´s southern and central regions have a higher burden than the north (HIV prevalence is 5.7% in the southwest; 7.2% in the south; 1.2% in the north; and 5.5% in the central region) [14]. The MoH has also received significant support from partners´ programs such as President's Emergency Plan for AIDS Relief (PEPFAR) program, and the U.S Centers for Disease Control and Prevention (CDC). While many studies have described the social behaviors and HIV epidemiology and behavioral practices in Cameroon, most of them focus on the general population or mother-to-child transmission. HIV among men in Chad is rarely addressed in the published literature. In this study, we conducted a comparative description of social behaviors and HIV risk factors among heterosexual men in the two neighboring central African countries - Cameroon and Chad - with contrasting HIV prevalence. The goal of this study was to explore behavioral factors associated with HIV among men in both countries that might explain the difference in the national HIV prevalence.

## Methods

### Setting and study design

The Republics of Chad and Cameroon are two neighboring countries in central Africa; Cameroon borders the southwest of Chad. Populations living on either side of the border between the northeastern region of Cameroon and southwestern regions of Chad have much in common: they share similar geographic and demographic distribution among the same ethnic groups. However, beyond the bordering regions, the rest of the two countries differ greatly, in terms of culture and geographic aspects. This was a retrospective cross-sectional study, in which we conducted quantitative analysis on a secondary dataset. We used data obtained from the demographic health survey (DHS): Chad DHS 2014-2015, and Cameroon DHS 2011 (https: // dhsprogram.com/). DHS surveys are designed to be nationally representative. Surveys were conducted in Chad and Cameroon among men and women aged 15-49 years old; however, our study includes only survey responses from men. The questionnaire included modules on sociodemographic information, HIV knowledge, sexual behavior, condom use, HIV testing, and sexually transmitted infections (STIs).

### Sample collection

In each country, DHS employed a stratified multi-stage cluster sample design in urban and rural areas. The first stage was identifying Enumeration Areas (EA). EA was drawn from census files. In the second stage, a sample of households was drawn from an updated list of households in each EA selected. In Chad, during the survey, enumerators interviewed men and women in 624 clusters in rural and urban areas. In total 17,233 households were surveyed (with a 99% response rate). Men were only interviewed in 1 in 3 of these households. Thus, 5248 men were interviewed (92% response rate). The survey was conducted from October 2014 to April 2015. In Cameroon, the survey was conducted in 578 clusters (urban and rural), in which 14,214 households were surveyed (with a 99% response rate). Men were interviewed in only 1 in 2 of these households. Thus, 7,191 men were interviewed (96% response rate). Data were collected from January to August 2011. Participants were asked whether they had ever been tested for HIV, if they used a condom during sex with their last partner, the number of sexual partners excluding their wife during the last 12 months, the total number of lifetime sexual partners, their educational level, their wealth index, their marital status, and history of STIs.

### Description of variables

Our two dependent variables were “ever been tested for HIV,” and “condom use during last sex with a most recent partner.” These variables were dichotomized into “Yes” or “No” based on the response to the question “Have you ever been tested for HIV?” and “did you use a condom during sex with your most recent partner?” respectively. Covariates were grouped into two categories consisting of HIV knowledge and risk factors. The sociodemographic characteristics included age, educational level, wealth index, marital status, and place of residence. The possible answers to the variable “Educational level” were coded “no education,” “primary,” “secondary,” and “higher;” however, we regrouped “no education” and “primary” into “primary or none”. We coded the wealth index as “lower” (for the poorest and poorer), “middle” (for middle), and “upper” (for richer and richest). Marital status was coded as “never in a union,” “married”, “living with a partner,” “widowed,” “divorced,” and “no longer living together/separated;” we reorganized the respondents into “never in a union,” “married/living with a partner,” “divorced/widowed/separated.” Continuous variables were categorized and proportions were compared using a chi-squared test.

### Statistical analysis

First, we computed the proportions of the sociodemographic characteristics in both countries for comparison (Table 1). Then, we fitted a separate multilevel logistic model for each country for the outcome variable “ever been tested for HIV” and “condom used during last sex with a most recent partner”. The two explanatory variables were used in interaction with sociodemographic covariates. The Estimated Odds Ratios (EOR) were obtained from the logistic regression models, and the 95% confidence interval. Chi-square was used for p-values (Table 2 and Table 3). All statistical analyses were performed using Statistical Analysis System software, SAS® 9.4 for Windows. A p-value of less than 0.05 was considered statistically significant. Microsoft Excel was used to display tables and graphs.

**Table 1:** sociodemographic distribution of Chadian and Cameroonian men, demographic and health survey (Chad DHS 2014-15, and Cameroon DHS 2011)

	Chad N (%)	Cameroon N (%)	Difference
**Population, N**	5248 (100)	7191 (100)	
**Age in year (median, IQR)**	30(20, 40)	28(20, 40)	2
**Educational level**			
Primary or less	3676 (70.05)	3053 (42.46)	27.59
Secondary	1400(26.68)	3558(49.48)	-22.8
Higher	172(3.28)	580(8.07)	-4.79
**Wealth index**			
Lower	1704 (32.47)	2419 (33.64)	-1.17
Middle	1099 (20.94)	1474 (20.50)	0.44
Upper	2445 (46.59)	3298 (45.86)	0.73
**Marital status**			
Never in union	1926 (36.70)	3187(44.32)	-7.62
Married/living with partner	3183 (60.65)	3635 (50.55)	10.1
Div/wid/sep	139 (2.65)	369 (5.13)	-2.48
**Type of place of residence**			
Urban	1515(28.87)	3626(50.42)	-21.55
Rural	3733(71.13)	3565(49.58)	21.55

Difference: quantitative difference between Chad and Cameroon data; Div/wid/sep: divorced, widowed or separated; IQR: interquartile range

**Table 2:** bivariate association between condom use and sociodemographic characteristic of Chadian and Cameroonian men, (Chad DHS 2014-15, Cameroon DHS 2011)

	Condom used during last sex with most recent partner							
	Chad				Cameroon			
	No N=3438 (93.35%)	Yes N=245 (6.65%)	EOR (CI)	P Value*	No N=3733 (67.47)	Yes N= 1800 (32.53)	EOR (CI)	p value*
**AGE group**								
15-19	143 (73.71)	51 (26.29)	1		178 (31.90)	380 (68.10)	1	
20-24	331 (77.88)	94 (22.12)	0.79(0.54, 1.2)	<.0001	356 (40.27)	528 (59.73)	0.69(0.55, 0.87)	<.0001
25-34	1051 (93.34)	75 (6.66)	0.2 (0.14, 0.3)	0.0047	1130 (66.31)	574 (33.69)	0.24(0.19, 0.29)	<.0001
>=35	1914 (98.71)	25 (1.29)	0.04 (0.02, 0.06)	<.0001	2069 (86.68)	318 (13.32)	0.07 (0.06, 0.08)	<.0001
**Educational level**								
Primary or less	2617 (98.31)	45 (1.69)	1		1928 (81.18)	447 (18.82)	1	
Secondary	717 (81.29)	165 (18.71)	13.38 (9.53, 18.8)	<.0001	1550 (58.34)	1107 (41.66)	3.08 (2.7, 3.5)	<.0001
Higher	105 (75)	35 (25)	19.4(11.96, 31.42)	<.0001	255 (50.90)	246 (49.10)	4.16 (3.4, 5.01)	<.0001
**Wealth index**								
Lower	1260 (97.60)	31 (2.4)	1		1534 (84.33)	285 (15.67)	1	
Middle	742 (97.25)	21 (2.75)	1.15 (0.65, 2.02)	0.0034	779 (69.24)	346 (30.75)	2.391 (2.0, 2.86)	0.0892
Upper	1437 (88.16)	193 (11.84)	5.46(3.71, 8.04)	<.0001	1420 (54.85)	1169 (45.15)	4.43 (3.82, 5.14)	<.0001
**Marital status**								
Never in union	331 (64.77)	180 (35.23)	1		516 (30.44)	1179 (69.56)	1	
Married/with partner	3054 (98.33)	52 (1.67)	0.03(0.02, 0.04)	<.0001	3023 (85.69)	505 (14.31)	0.07(0.06, 0.08)	<.0001
div/wid/sep	54 (80.60)	13 (19.40)	0.44 (0.24, 0.83)	0.0042	194 (62.58)	116 (37.42)	0.26 (0.20, 0.34)	0.7895
**Place of residence**								
Urban	803 (82.36)	172 (17.64)	1		1559 (56.20)	1215 (43.80)	1	
Rural	2636 (97.31)	73 (2.69)	0.13 (0.01, 0.17)	<.0001	2174 (78.80)	585 (21.20)	0.34 (0.30, 0.38)	<.0001

EOR: estimated odds ratio; CI: 95% confidence interval; Div/wid/sep: divorced, widowed or separated; *: Chi-square

**Table 3:** bivariate association between HIV test and sociodemographic characteristics of Chadian and Cameroonian men, (Chad DHS 2014-15, Cameroon DHS 2011)

	Ever been tested for HIV							
	Chad				Cameroon			
	No N=4593 (87.55%)	Yes N=653 (12.45%)	EOR (95% CI)	P value	No N=4187 (58.23)	Yes N=3004 (41.77)	EOR (95% CI)	p value
**Age group**								
15-19	1191(97.46)	31(2.54)	1		1377(85.42)	235(14.58)	1	
20-24	624(84.90)	111(15.10)	6.84 (4.54, 10.3)		717(60.20)	474(39.80)	3.88 (3.23, 4.64)	0.1133
25-34	1027 (82.16)	223 (17.84)	8.34(5.68, 12.26)		843 (45.49)	1010(54.51)	7.02(5.95, 8.28)	<0.0001
>=35	1751 (85.88)	288 (14.12)	6.32(4.33, 9.22)		1250(49.33)	1285 (50.67)	6.02 (5.14, 7.06)	<0.0001
**Educational level**								
Primary or less	3472 (94.48)	203 (5.52)	1		2228(72.98)	825(27.02)	1	
Secondary	1056 (75.48)	343 (24.52)	5.56(4.6, 6.7)	0.6646	1851(52.02)	1707(47.98)	2.5 (2.25, 2.77)	<0.0001
Higher	65 (37.79)	107 (62.21)	28.14(20.05, 39.5)	<0.0001	108 (18.62)	472(81.38)	11.8 (9.44, 14.77)	<0.0001
**Wealth index**								
Lower	1574 (92.37)	130 (7.63)	1		1866 (77.14)	553 (22.86)	1	
Middle	1026 (93.44)	72 (6.56)	0.85 (0.6, 1.15)	<0.0001	883 (59.91)	591 (40.09)	2.26(1.2, 2.6)	0.2013
Upper	1993 (81.55)	451 (18.45)	2.74 (2.23, 3.37)	<0.0001	1438 (43.60)	1860 (56.40)	4.37(3.89, 4.9)	<0.0001
**Marital status**								
Never in union	1735(90.18)	189(9.82)	1		2224(69.78)	963(30.22)	1	
Married/with part	2759 (86.68)	424 (13.32)	1.41 (1.18, 1.7)	0.0062	1770 (48.69)	1865 (51.31)	2.44 (2.2, 2.69)	<0.0001
div/wid/sep	99 (71.22)	40 (28.78)	3.71(2.5, 5.52)	<0.0001	193 (52.30)	176 (47.70)	2.1 (1.7, 2.62)	0.0052
**Place of residence**								
Urban	1119(73.91)	395(26.09)	1		1745(48.12)	1881(51.88)	1	
Rural	3474(93.09)	258(6.91)	0.21 (0.18, 0.25)	<0.0001	2442(68.50)	1123(31.50)	0.427 (0.39, 0.5)	<0.0001

EOR: estimated odds ratio; CI: 95% confidence interval; *: Chi-square

### Ethics

DHS questionnaires and procedures were reviewed and approved by the ICF International Institutional Review Board (IRB) and by each host country IRB (see more at http: //dhsprogram.com/). Besides, we submitted a research application to the Georgia State University Review Board and we obtained an IRB waiver (IRB Number: H17467, Reference Number: 343492).

## Results

### Sociodemographic characteristics of the participants

In Chad, 5,248 participants were included in the study, and the median age of respondents was 30 (IQR, 20-40) years. In Cameroon, the sample size was 7, 191, and the median age was 28 (IQR, 20-40). Most of the participants (70.05%) in Chad had only primary education or none, compared to 42.46% in Cameroon. Almost half of the participants (49.58%) in Cameroon lived in rural areas, versus 71.1% of Chadians. 60.65% of participants in Chad were married or living with a partner, compared to 50.55% in Cameroon and the median age was similar in both countries. The wealth index was similar in both countries, at all levels as shown in Table 1.

### HIV knowledge and risk factors distribution among participants

HIV testing was very low in Chad, only 12.45% of participants reported the previous testing for HIV; in Cameroon, 41.77% had been tested before. In Chad, 46.40% of participants did not know where to get HIV testing, compared to 11.15% in Cameroon. The median number of lifetime sexual partners was 2 (IQR, 1-4) in Chad, and 6 (IQR, 3-15) in Cameroon. Among participants, 31.63% in Chad versus 10.76% in Cameroon had only 1-lifetime sexual partner. In Chad, 86.95% of participants reported having no sex partner other than their spouse in the last 12 months; in Cameroon, the proportion was 57.3%. Among the respondents in Chad and Cameroon, 6.65% and 32.53% respectively reported using a condom during sex with their most recent partner, and 54.49% and 11.85% respectively in Chad and Cameroon did not know any source for condoms. 1.1% of Chadians reported an STI in the last 12 months compared to 3.98% in Cameroon.

### Bivariate association between condom use and sociodemographic characteristics

As shown in Table 2, 6.65% and 32.53% of participants in Chad and Cameroon have used condoms during the last sex with most recent partners. The age group 15-19 years had the highest condom use during last sex in both countries; the proportion was 26.29%, with an estimated odds ratio (EOR) of 1 (ref) in Chad; in Cameroon, the proportion was 68.10% with an EOR of 1 (ref). In both countries, participants with an upper level of wealth index had the highest rate of condom use with the most recent partner, 11.84% with an EOR of 5.46(3.71, 8.04) in Chad; and 45.15% with an EOR of 4.43(3.82. 5.14) in Cameroon. There was a significant difference between those that reported “Yes” and “No” (p-value < 0.001) among this group in both countries. People that reported “never been in union” in both countries had the highest rate of condom use with the most recent partner (35.23% in Chad and 69.56% in Cameroon). Urban respondents in both countries were more likely to use condoms with their most recent partner compared to people living in the rural areas (17.64% in Chad and 43.80% in Cameroon).

### Bivariate association between HIV test and sociodemographic characteristics

Table 3 shows that the proportion of people who reported that they had ever been tested for HIV increased with age in both countries. The highest rate of HIV testing was in the age group 25-34 years (17.84% with EOR 8.34(5.68, 12.26) in Chad, and 54.51% with EOR 7.02 (5.95, 8.28) in Cameroon. The lowest rate of “ever been tested for HIV” was observed among individuals with primary or less educational level in both countries (5.52% with EOR 1 (ref) in Chad), and 27.02% with an EOR 1 (ref) in Cameroon. Additionally, the highest proportion of people who reported “ever been tested” was observed among individuals with upper-level wealth index in both countries (18.45% with EOR 2.74 (2.23, 3.37) in Chad, and 56.40% EOR 4.37(3.89, 4.9) in Cameroon). Chadian men who were divorced, widowed, or separated had the highest HIV testing (28.78% with EOR 3.71(2.5, 5.52)), however, Cameroonian men who were married or living with a partner had the highest HIV testing with 51.31% EOR 2.44 (2.2, 2.69). More participants in urban areas reported receiving HIV testing than rural residents; 26.09% and 51.88% among urban residents in Chad and Cameroon respectively.

## Discussion

We observed that the age distribution is similar among participants in Chad and Cameroon. However, there was a significant difference in educational level among participants, with a very low level in Chad, where 70.05% of the respondents had only primary education or none, compared to 42.46% in Cameroon. This difference is also evident in the adult literacy rate [15]. The difference in education levels is proportional to the HIV screening (41.77% in Cameroon and 12.45% in Chad). In both countries, participants with low levels of education and literacy were less likely to receive HIV screening. Several studies have shown an association between education level and HIV testing among men in other countries [16, 17]. Even though Cameroon is relatively wealthier than Chad, our results show that the participants´ wealth index was similar in both countries, at all levels.

Also, we found that a high percentage (46.40%) of Chadian men did not know where to get HIV testing, which may partially explain the low testing rate. In Chad, the majority of testing centers are in urban cities; however, most Chadian men (71.1%) live in rural areas. Moreover, the majority of testing centers are linked to antenatal services and prevention of mother-to-child transmission of HIV (PMTCT) programs, which do not include men the majority of the time. Nonetheless, previous analysis using DHS data showed an even lower proportion (7.04%) of HIV testing among men in Chad [18]. HIV testing is essential to HIV treatment and attainment of the UNAIDS goals; these findings suggest that both countries need to implement new methods to reach out to men for HIV testing, for example, self-testing, work-place testing, mobile testing, etc. Higher HIV testing coverage in Cameroon could be explained by the fact that the MoH benefits from the support of the international partnership of the President's Emergency Plan for AIDS Relief (PEPFAR) program, and the U.S Centers for Disease Control and Prevention (CDC), which do not have HIV/AIDS programs in Chad.

**Figure 1:**
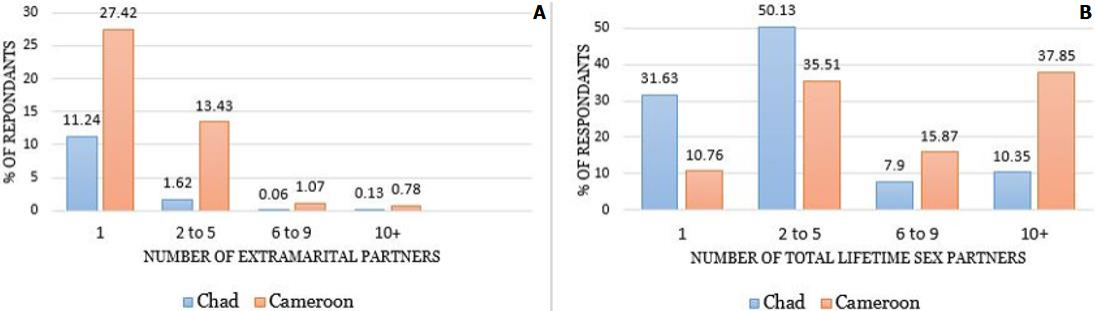
self-reported extramarital partners (A) and total lifetime (B) sexual partners among men in Chad and Cameroon

Results show that Chadian men have a lower number of lifetime sexual partners (median 2, IQR, 1-4) compared to Cameroonian (median 6, IQR 3-15) and a substantial proportion of Chadian men (86.96%) reported fidelity to their domestic partners in the last twelve months compared to 57.30% of Cameroonian men. Even though, in general, Cameroonian men were more knowledgeable about HIV than Chadian men, the comparatively higher number of non-marital sexual partners among Cameroonian men seems to be the main factor in the higher HIV prevalence (Figure 1); albeit, this result is based on self-reported information. There is a need for more emphasis on HIV education among Chadian men, which has the potential to substantially reduce HIV prevalence and incidence. Other factors that may explain the lower number of non-marital sexual partners among Chadian men are the dominant traditional cultural norms in rural areas where commercial sex work and multiple sexual partners are censured by the tradition, and early marriage is very frequent [19]. A program aiming at reducing the number of sexual partners could be effective in reducing HIV prevalence in Cameroon.

Condom use with the most recent partner was low in both countries, respectively 6.65% and 32.53% in Chad and Cameroon. The highest rate of condom use was observed among men that reported never having been in a union in both countries but still substantially low in Chad. The proportion was very low among rural respondents in Chad. These results show that condom distribution programs should also target rural areas where the majority of Chadians live. The wealthiest respondents in both countries had the highest condom use, probably because of their correlation with urban residencies where access to condoms is easier compared to the provinces. The MoH in both countries could strategize on implementing new methods of condom distribution, including in workplaces and communities.

### Strengths and limitations

Our study has several limitations. First, as the data were obtained from the DHS survey, we conducted a cross-sectional study from which we could not make a causal inference about our findings. Second, participants in this survey self-reported sexual behavior in the communities where open discussion about sex is still taboo; therefore it is difficult to eliminate some biases such as under-reporting or over-reporting. Third, we did a comparative analysis between two neighboring countries using data that were not collected during the same timeframe, albeit the methodology was the same. Moreover, HIV programs in these countries are changing constantly and the findings from earlier surveys might not represent today´s statistics. Finally, in this study, we defined our outcome variable as “condom use during last sex with a most recent partner.” This does not allow us to capture the overall correct and consistent use of condoms in the two countries. Nonetheless, our study used the DHS data, which is representative of diverse populations at the national and sub-national levels. Surveys were conducted in all regions of both countries.

### The implication for public health policies

Our study has several implications for health policies. First, our findings suggest that there is a crucial need to encourage HIV screening and testing among Chadian men, especially in rural areas. Testing also needs to be increased in Cameroon to meet the first UNAIDS target. The MoH in both countries should focus more on HIV literacy among rural communities, and the program should encourage men to participate in antenatal visits with their wives because HIV counseling and screening is already incorporated in the antenatal visits in both countries, and men are allowed to participate. In Cameroon, the dominant risk factor for HIV was multiple sexual partners. There is a need for further research on this subject in Cameroon and for increased efforts to reduce multiple concurrent partnerships and/or reduce the risk of those partnerships through consistent condom use and timely initiation of ART for treatment-as-prevention in the communities.

## Conclusion

Condom use and HIV testing rates were very low among Chadian men compared to Cameroonian men and were positively associated with education level and wealth index in both countries. Also, HIV knowledge was higher among respondents in Cameroon than in Chad. However, Cameroonian men were more likely to have multiple sexual partners and extra-marital relationships than Chadian men. Government and partners could support more research and campaigns that aim at reducing multiple sexual partnerships - and/or HIV risk within those partnerships via condoms and HIV Treatment as Prevention - among the communities in Cameroon.

### What is known about this topic

- Cameroon has one of the highest HIV prevalence in Central Africa;
- Chad has a very low HIV testing rate;
- Chad has lower HIV prevalence and incidence comparing to Cameroon.

### What this study adds

- This study discloses that in Cameroon, the dominant risk factor for HIV was multiple sexual partners and extra-marital relationships (in comparison to neighbor Chad);
- In Chad, men have a lower number of total lifetime sexual partners and more likely to be loyal to their domestic partners comparing to Cameroonian men;
- Both countries unlikely to meet the first UNAIDS target (by 2020, 90% of people living with HIV will know their status).
